# Capsid-Targeting Biologic Achieves Broad HIV-1 Neutralization with a High Barrier to Resistance

**DOI:** 10.3390/ijms27156883

**Published:** 2026-08-01

**Authors:** Florence M. Stel, Esther M. Zijlstra-Willems, Ad C. van Nuenen, Brigitte D. M. Boeser-Nunnink, Teunis B. H. Geijtenbeek, Neeltje A. Kootstra

**Affiliations:** 1 Department of Experimental Immunology, Amsterdam UMC Location AMC, University of Amsterdam, 1105 AZ Amsterdam, The Netherlands; f.m.stel@amsterdamumc.nl (F.M.S.);; 2 Amsterdam Institute for Immunology and Infectious Diseases, 1105 AZ Amsterdam, The Netherlands

**Keywords:** anti-capsid biologic, HIV-1, nanobody, viral escape

## Abstract

Antiretroviral therapy (ART) has proven effective in suppressing HIV-1 replication, but further development of HIV-1 inhibitors is continually driven by the challenge of drug resistance and viral adaptation. The HIV-1 capsid is a promising target for treatment due to its high sequence conservation as well as its crucial role in the viral life cycle. Recently, we have developed a novel capsid-targeting biologic that prevents HIV-1 replication by efficient degradation of newly synthesized capsid. Here, we have investigated the sensitivity to viral escape as well as the breadth of this biologic against HIV-1 subtypes. The capsid-targeting biologic efficiently blocked replication of different primary HIV-1 isolates, and continuous exposure of these viruses to the biologic resulted in viral breakthrough of two out of ten primary HIV-1 isolates tested. Notably, the breakthrough variants did not have amino acid changes in the nanobody epitope but primarily in the matrix region. The breakthrough variants remained sensitive to the biologic albeit to a lesser extent. In the absence of the biologic, breakthrough variants showed increased replication kinetics when compared to their parental virus, suggesting that adaption to the biologic is likely due to the increased viral production and that the target area of the biologic is too conserved for actual escape. This is further underscored by the broad specificity of the biologic as importantly the biologic blocked infection of different HIV-1 subtypes that occur worldwide (A, B, C, D, CRF01_AE, CRF02_AG). These results demonstrate the broad neutralization potential of anti-capsid biologics with a high barrier to resistance, making capsid-targeting inhibitors important for novel antiretroviral drug strategies worldwide.

## 1. Introduction

HIV-1 antiretroviral therapy (ART) has significantly improved life expectancy for people with HIV-1 (PWH). However, the emergence of drug-resistant variants is still a major challenge [1]. Group M, the prevalent HIV-1 group, consists of 10 subtypes (A–D, F–H, J–L), eight sub-subtypes, and a large number of circulating recombinant forms (CRFs) [2,3]. These different subtypes have a distinct geographical distribution, with subtype A being highly prevalent in Eastern Europe and Central Asia, subtype B in Western and Central Europe, as well as Latin America, whereas subtype C is found mostly in Southern Africa and India [3,4]. Moreover, different subtypes exhibit distinct drug resistance patterns. These differences in drug resistance patterns lead to reduced efficacy of ART and complicate treatment strategies [4,5,6,7]. One HIV-1 protein that has shown less sensitivity to resistance mutations is the capsid protein (CA). This protein, part of the polyprotein Gag, is highly conserved across both subtypes and HIV-1 groups [8,9] and has therefore emerged as an important target for the development of novel antiretrovirals [10,11]. Specifically, sequences important for the structure and function of CA show little variation, including helix 9 at the CA dimer interface and the major homology region, with the latter remaining conserved even among other retroviruses [12,13].

Another characteristic of CA that strengthens its potential as a broad-spectrum treatment target is that it plays a crucial role in both early and late stages of the viral life cycle [14,15]. During the early stages of infection, CA not only transfers the viral RNA to the nucleus and protects the viral genome against nucleic acid sensors and restriction factors in the cytosol, but also facilitates reverse transcription and nuclear import [16]. At later stages, CA plays a critical structural role in organizing the assembly, budding, and maturation of new virions. CA interacts with various host factors by recruiting or even mimicking host proteins to promote viral replication or to evade host restriction factors that would inhibit replication, further demonstrating that CA is an active and integral part of HIV-1 replication [17,18,19,20].

Because of this complex and balanced nature of CA structure and its role in viral fitness, mutations often result in viral attenuation or replication-deficient virus, further supporting CA as a target for antiretroviral drugs [21,22,23]. Potential anti-capsid drugs have been investigated for years, resulting in a large repertoire of compounds targeting various components of CA [11]. Currently, the only inhibitor approved by the FDA is lenacapavir (LEN, GS-6207). This long-acting small molecule inhibitor binds to CA monomers and is effective across a range of HIV-1 subtypes, regardless of treatment history [24,25,26]. However, like many antiretroviral drugs, resistance to LEN has appeared, both in viral breakthrough assays and in participants of LEN clinical trials [25,26,27,28,29]. Key resistance-associated mutations in Gag have been identified, with N74D and Q67H emerging as the main mutations [30,31,32], showing up during virological failure in vivo [28,33]. Therefore, research progress is being made on the production of next-generation compounds to address these challenges, as well as improve factors like oral bioavailability. Moreover, combinations with other antiretroviral agents are explored as potential therapeutic strategies [34]. We have recently developed another potential capsid inhibitor in the form of a nanobody-based biologic [35]. This biologic aCA-Fc consists of a CA-targeting nanobody fused to the Fc domain of human IgG1 and targets newly produced Gag in the later stage of viral replication. The Gag/CA-biologic complex is recognized by tripartite motif 21 (TRIM21), a high-affinity intracellular Fc receptor that plays a role in intracellular antiviral immunity [36,37]. Upon TRIM21 binding to the Fc domain, the Gag/CA-biologic complex is directed to the proteasome, resulting in degradation of Gag/CA and inhibition of viral replication [35].

In this study we aimed to further investigate the therapeutic potential of capsid-targeting biologic aCA-Fc by examining its neutralization capacity and barrier to resistance. We observed limited emergence of variants that were able to replicate in presence of the biologic, indicating a high genetic barrier to resistance. The breakthrough variants remained sensitive to aCA-Fc but overcame aCA-Fc pressure by increased replication and virus production. Importantly, our data show that aCA-Fc is very effective against different HIV-1 subtypes occurring worldwide, including subtypes B and C, further underscoring its broad applicability and complementarity to current therapies.

## 2. Results

### 2.1. Adaptation of HIV-1 to the aCA-Fc Biologic by In Vitro Evolution

**Figure 1:**
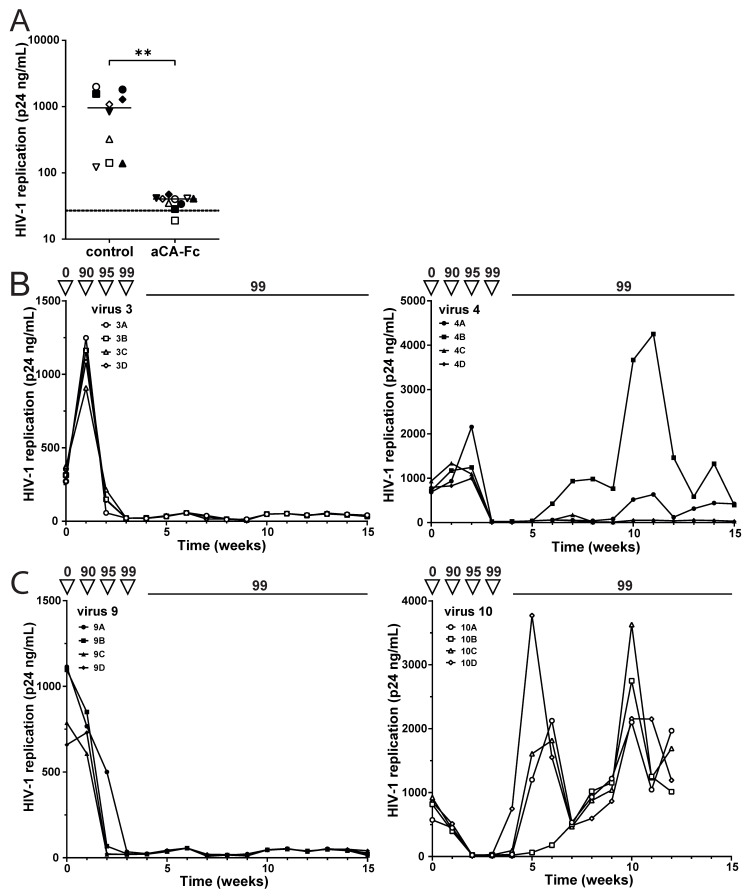
Adaptation to aCA-Fc restriction is rare in *in vitro* evolution of primary HIV-1 isolates. (**A**) U87.CD4.CCR5 cells expressing aCA-Fc or an empty vector control were inoculated with ten different CCR5-using primary HIV-1 variants (at MOI 0.01) previously isolated from five different PWH. Viral replication was determined after seven days by measuring Gag/CA production in the culture supernatant using p24 ELISA. The symbols represent the mean of two or three independent experiments, different symbols represent different isolates, and the dotted line indicates the assay background level. Statistical analysis was performed using a paired *t*-test. ** *p* < 0.01. (**B**,**C**) Replication kinetics of primary CCR5 variants from PWH-2 (**B**) and PWH-5 (**C**) during in vitro culturing in U87.CD4.CCR5 cells with increasing percentages of aCA-Fc-producing cells. The arrows on top indicate the percentage of U87.CD4.CCR5 cells with aCA-Fc. The right panel shows the breakthrough infection, whereas the left panel shows complete inhibition of a CCR5 variant obtained from the same PWH.

We explored the ability of aCA-Fc to inhibit replication of ten primary CCR5-using HIV-1 isolates previously isolated from five PWH of the Amsterdam Cohort Studies on HIV infection and AIDS (ACS) [38,39]. We used U87.CD4.CCR5 cells stably expressing the aCA-Fc biologic (U87.CD4.CCR5-aCA-Fc) that have previously been constructed using a lentiviral vector [35]. Through a binding ELISA we determined that these cells on average express 55.6 ± 18.6 pg (mean ± SD) aCA-Fc per cell. U87.CD4.CCR5-aCA-Fc and U87.CD4.CCR5 cells were inoculated with the different isolates at a MOI of 0.01 to confirm the replication capacity of these isolates in these cell lines. After seven days, replication was determined by measuring Gag/CA using p24 ELISA. Viral replication of all ten primary isolates was observed in U87.CD4.CCR5 cells without the biologic, whereas replication was inhibited to (near) background levels in U87.CD4.CCR5-aCA-Fc (Figure 1A). Using these isolates, we investigated whether HIV-1 can adapt to aCA-Fc restriction in an *in vitro* evolution assay where virus is grown under increasing selection pressure of aCA-Fc. For each virus, four parallel U87.CD4.CCR5 cultures were inoculated. HIV-1 replication was detected in all cultures after one to three weeks, and these culture supernatants were then used to inoculate a mix of U87.CD4.CCR5 wildtype cells and those expressing aCA-Fc. It was previously shown that expression of aCA-Fc did not affect cell viability and no differences in morphology and growth rate were observed during the culturing of these cell lines [35], and over time the proportion of aCA-Fc-expressing U87.CD4.CCR5 cells was increased (Figure 1B,C). In all cultures, viral replication decreased to background Gag/CA levels within two weeks when cells expressing aCA-Fc had been increased to >90%. Viral replication of all variants remained undetectable or very low for at least two consecutive weeks during which the proportion of aCA-Fc-expressing cells reached 99% (Figure 1B,C and Figure S1). Notably, breakthrough of viral production was observed for two viral variants (virus 4 and 10) obtained from two different PWH. For virus 10, replication increased above the detection limit in all four parallel cultures (subsequently referred to as 10A, 10B, 10C and 10D) between week four and six, indicative of viral adaptation. For virus 4, Gag/CA production became detectable in two out of four parallel cultures (subsequently referred to as 4A and 4B) between week six and ten. Taken together, our data show viral adaptation to aCA-Fc restriction for two out of the ten primary variants.

### 2.2. Adaptation of HIV-1 to aCA-Fc Is Not Associated with Mutations in Capsid

**Figure 2:**
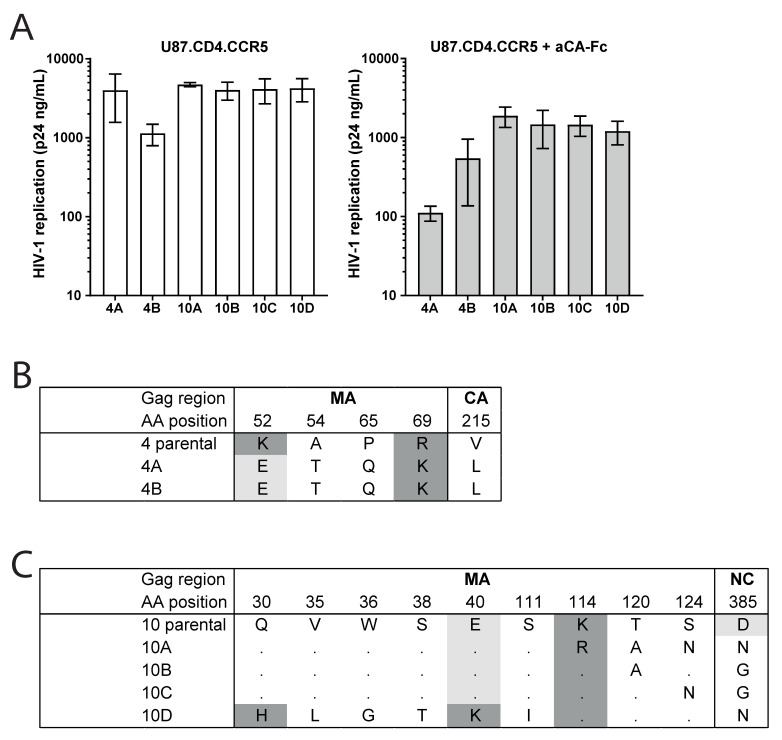
Breakthrough variants remain sensitive to aCA-Fc. (**A**) Breakthrough variants were used to inoculate cultures with wildtype (white bar) or aCA-Fc-producing (grey bar) U87.CD4.CCR5 cells for seven days. Viral replication is determined by measuring Gag/CA production in the culture supernatant using p24 ELISA. Bars represent mean + SEM of two or three independent experiments. (**B**,**C**) Breakthrough variants from parental virus 4 (**B**) and virus 10 (**C**) were sequenced and amino acid (AA) changes respective to the parental variant are shown. AA positions are mapped to consensus HXB2D sequence and relative to the region of Gag (MA/CA/NC); the dots indicate no AA change compared to the parental virus; negatively charged AA (D/E): light grey; positively charged AA (K/R): dark grey. MA: matrix; CA: capsid; NC: nucleocapsid.

To examine whether breakthrough viral isolates 4A–B and 10A–D had adapted to aCA-Fc, infection of the breakthrough viruses was determined in U87.CD4.CCR5 cells with and without aCA-Fc. After seven days, all six breakthrough viral variants were able to replicate in both U87.CD4.CCR5-aCA-Fc and U87.CD4.CCR5 cultures (Figure 2A) as demonstrated by Gag/CA production in the culture supernatant. Notably, replication of the breakthrough variants was less efficient in the presence of aCA-Fc than without aCA-Fc, suggesting that the breakthrough variants are still sensitive to aCA-Fc restriction.

To determine whether viral adaptation to aCA-Fc was associated with the emergence of mutations in capsid, sequence analysis of the *gag* region was performed (Figures S2 and S3). *In vitro* evolution did not show any amino acid changes in the aCA-Fc epitope in capsid (CA) of breakthrough variants (AA196–205 and 211–218 relative to the start of CA) as compared to their parental variant (Figure S2). Parental variants were sequenced from primary isolates that were grown on wildtype U87.CD4.CCR5 and used to inoculate at the start of in vitro evolution. Variants 4A and 4B showed identical amino acid changes in the MA region 52–69 (K52E, A54T, P65Q and R69K) of which the K52E change led to a charge change from positive to negative, in combination with the V83L amino acid change in CA (Figure 2B). Breakthrough variants derived from parental virus 10 showed variable amino acid changes, ranging from two to seven amino acid changes located in the matrix (MA) and nucleocapsid (NC) (Figure 2C). Amino acid changes in MA were observed in two regions (AA30–40 and AA111–124) and position 8 of NC: variants 10A-C contained amino acid changes in MA AA114–124 in combination with an amino acid change at NC position 8 (variant 10A: K114R/T120A/S124N in MA and D8N in NC; variant 10B: T120A in MA and D8G in NC; variant 10C: S124N in MA and D8G in NC) of which the amino acid change in NC led to a charge change from negative to neutral. Variant 10D contained amino acid changes in two regions in MA (Q30H/V35L/W36G/S38T/E40K/S111I), of which the E40K change led to a charge change from negative to positive. Our data indicate that depending on the viral sequence backbone, HIV-1 can adapt to replicate in the presence of aCA-Fc by the introduction of amino acid changes mainly in MA but outside of the capsid region targeted by the biologic.

### 2.3. aCA-Fc Breakthrough Viral Variants Exhibit Improved Replication Kinetics

**Figure 3:**
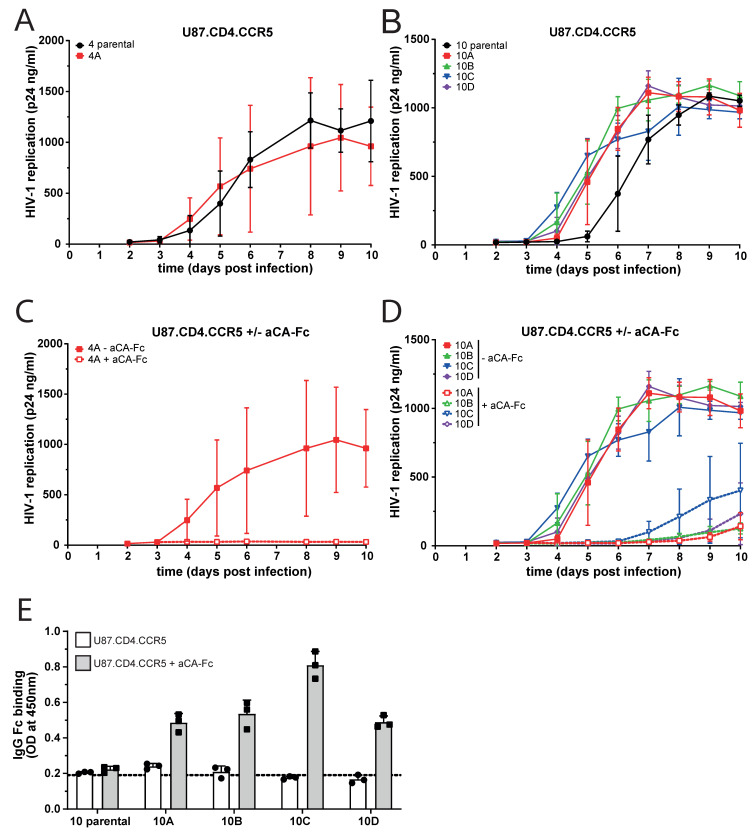
Increased replication kinetics provide breakthrough of HIV-1 variants. (**A**,**B**) Wildtype U87.CD4.CCR5 cells were inoculated with 100 TCID50 units of breakthrough or parental virus 4 (**A**) or virus 10 (**B**). Viral replication was determined over time by measuring the amount of Gag/CA in the supernatant using p24 ELISA. (**C**,**D**) U87.CD4.CCR5 with (dashed lines) and without (solid lines) aCA-Fc were inoculated with 100 TCID50 units of breakthrough or parental virus 4 (**C**) and virus 10 (**D**). (**E**) For virus 10, the presence of virion-associated aCA-Fc after nine days was determined by ELISA detecting IgG-specific Fc, where the dotted line indicates the assay background level. The graphs represent the mean for three independent experiments; the bars represent mean + SD.

As the breakthrough variants were still sensitive to aCA-Fc restriction, we next assessed whether the observed amino acid changes affect viral fitness of the viruses. Therefore, U87.CD4.CCR5-aCA-Fc and U87.CD4.CCR5 were inoculated with 100 TCID_50_ of the parental or the breakthrough variant, and viral replication was determined by the production of Gag/CA by p24 ELISA. For breakthrough virus 4B, we did not manage to acquire enough replication-competent virus for further experiments, most likely due to its attenuation. Breakthrough variant 4A showed similar growth compared to the parental variant (Figure 3A) and no growth in the presence of aCA-Fc at the chosen starting inoculation of 100 TCID_50_ units (Figure 3C), which indicates that replication of this variant is still restricted by aCA-Fc. All breakthrough viruses from parental virus 10 showed increased replication kinetics in U87.CD4.CCR5 cells compared to their respective parental variant (Figure 3B), as the time to reach 50% of the maximal Gag/CA production (t_50_) was lower for breakthrough variants (t_50_ was 6.4 days for parental virus 10 and 5.1, 5.0, 4.6 and 5.1 days for breakthrough variants 10A-D respectively). Around day 8 the Gag/CA level plateaued for all viruses. The breakthrough viruses exhibited less replication capacity in U87.CD4.CCR5 expressing aCA-Fc, as compared to U87.CD4.CCR5, and reached lower levels of Gag/CA by day 10 (Figure 3D). However, on day 10, Gag/CA production could be detected for all breakthrough variants, whereas no replication of the parental viruses could be detected over the course of infection. Moreover, Gag/CA-bound aCA-Fc could be detected in the supernatant of infected cells (Figure 3E), demonstrating that the biologic was still bound to Gag/CA in the produced virions. These results suggest that improved replication kinetics could serve as a potential mechanism through which breakthrough viruses from parental virus 10 could achieve virus adaptation to aCA-Fc restriction.

### 2.4. The aCA-Fc Biologic Degrades Gag with LEN-Resistant Mutations

**Figure 4:**
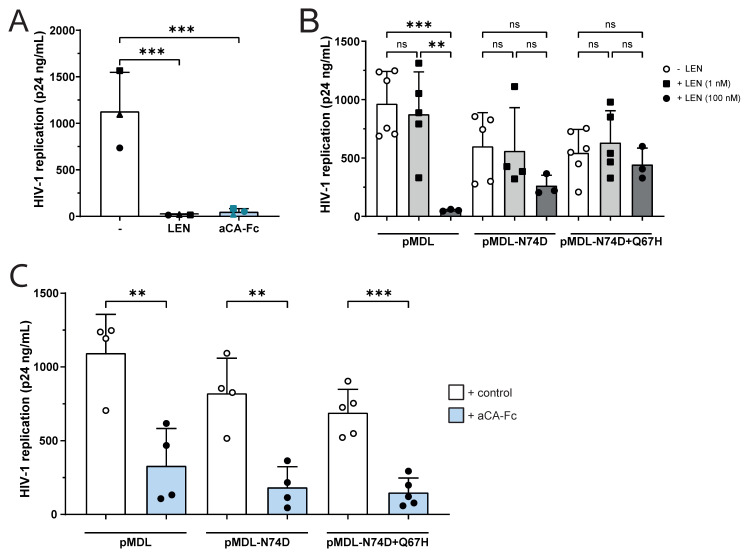
aCA-Fc blocks production of LEN-resistant mutants. (**A**) U87.CD4.CCR5 cells were incubated for 30 min with LEN (1 nM) and in parallel with aCA-Fc-producing U87.CD4.CCR5 cells inoculated with NL4-3-Ba-L (MOI 0.1). Viral replication was determined after seven days by measuring the amount of Gag/CA in the supernatant using p24 ELISA. (**B**,**C**) HEK293T cells were co-transfected with Rev- and GagPol-expressing constructs (pRSV-Rev and pMDL) containing LEN-resistant single (N74D) and double (N74D + Q67H) mutations. LEN was added at 1 nM or 100 nM final concentration (**B**) or co-transfected with aCA-Fc (**C**). Gag production was determined three days after transfection of pMDL [35], pMDL-N74D or pMDL-N74D + Q67H in HEK293T using p24 ELISA of supernatant. Symbols represent independent experiments; bars represent mean + SD. Statistical analysis was performed using a two-way ANOVA with the Šidák multiple comparisons test (**A**,**B**) or the paired *t*-test (**C**). ns = not significant, *** *p* < 0.001, ** *p* < 0.01.

Next, we investigated the efficacy of the aCA-Fc biologic against known resistance mutations of capsid inhibitor lenacapavir (LEN) [25,26,27,31]. The addition of LEN to U87.CD4.CCR5 cells inoculated with NL4-3-Ba-L blocked HIV-1 replication, which was also the case if the cells were expressing aCA-Fc (Figure 4A). As aCA-Fc degrades newly produced Gag/CA [35], we transfected HEK293T cells with GagPol overexpression construct containing LEN resistance mutations N74D and Q67H, and Gag/CA production was measured in the culture supernatant by p24 ELISA. Addition of LEN at high concentration efficiently decreased wildtype Gag but not Gag with resistance mutations N74D and Q67H (Figure 4B). Notably, co-transfection of aCA-Fc resulted in a significant decrease in both wildtype and LEN-mutant Gag production (Figure 4C). These results show that the aCA-Fc biologic recognizes and degrades LEN-resistant Gag mutants.

### 2.5. The aCA-Fc Biologic Blocks a Wide Variety of HIV-1 Subtypes from Around the World

**Figure 5:**
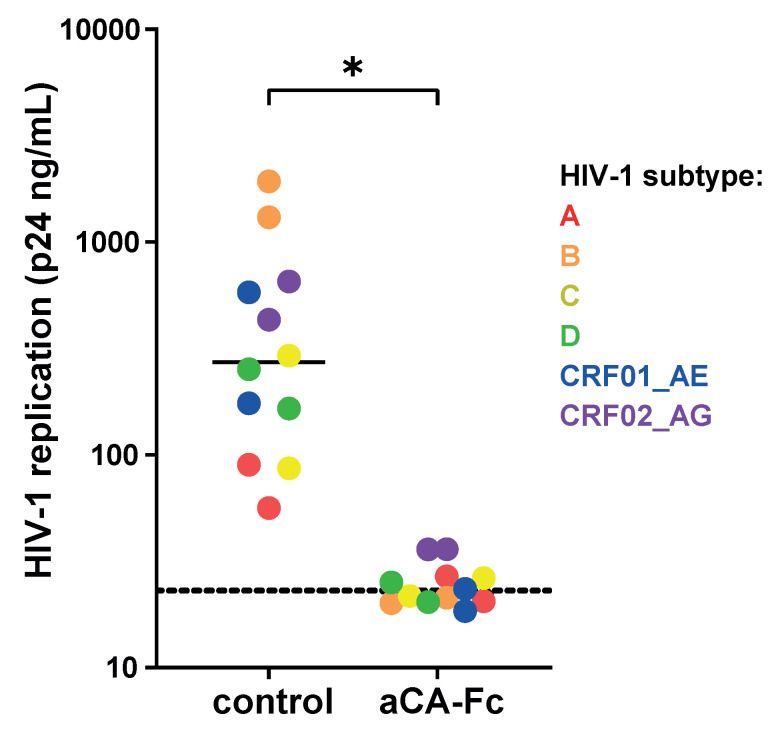
aCA-Fc effectively blocks replication of HIV-1 subtypes occurring worldwide. U87.CD4.CCR5 cells stably expressing aCA-Fc or an empty vector control were infected with different HIV-1 subtypes (A, B, C, D, CRF01_EA, CRF02_AG) at a MOI of 0.01 or 0.05. Viral replication was determined seven days after infection by measuring Gag/CA in supernatant through p24 ELISA. For each HIV-1 subtype two variants were included: HIV-1 subtype A 99KE_KNH1088 and 00KE_KSM4030; subtype B 91US_4 and 92FR_BX08; subtype C 94IN_20635-4 and 00TZ_A125; subtype D 98UG_57128 and 00KE_NKU3006; subtype CRF01_AE 90TH_CM235 and 96TH_NI1046; subtype CRF02_AG 91DJ_263 and 01CM_0008BBY. The symbols represent the mean of the three independent experiments, and the dotted line indicates the assay background level. Statistical analysis was performed using a paired *t*-test. * *p* < 0.05.

Amino acid variation between different HIV-1 subtypes was observed in MA, CA and NC, with CA exhibiting the least amount of variation across subtypes (Figure S4). Some amino acid substitutions in the aCA-Fc epitope in the CA region were present at position 198 and 202 (lysine (K)/arginine (R)), position 199 (threonine (T)/serine (S)) and position 210 (leucine (L)/isoleucine (I)). To investigate whether these variations were able to support viral replication in the presence of aCA-Fc, we determined the neutralization capacity of the aCA-Fc biologic against various HIV-1 subtypes (Figure 5). For each HIV-1 subtype two different variants were included. U87.CD4.CCR5-aCA-Fc and U87.CD4.CCR5 were inoculated with the different HIV-1 subtypes at a MOI of 0.01 and 0.05. Seven days after infection, replication was determined by measuring Gag/CA using p24 ELISA. Strikingly, replication of all subtypes decreased to background levels in the presence of aCA-Fc, whereas replication to varying degree was observed in U87.CD4.CCR5 without the biologic. These data strongly suggest that aCA-Fc has a high breadth in suppressing viral replication across genetically distinct HIV-1 subtypes worldwide.

## 3. Discussion

The HIV-1 capsid (CA) has emerged as a promising target for new antiretroviral drugs, as it plays a critical role at multiple stages of the HIV-1 replication cycle and is therefore highly conserved. We have recently developed an anti-capsid biologic aCA-Fc that blocks replication by degrading newly synthesized CA [35]. Here, we investigated the sensitivity to viral escape as well as the breadth of aCA-Fc restriction. Pressure from this restriction led to the breakthrough of six viruses out of a total of forty tested, suggesting that escape is very limited. Strikingly, these breakthrough variants did not have any mutations in the capsid region targeted by aCA-Fc. Breakthrough variants were still sensitive to aCA-Fc restriction but managed to replicate at a low level. The breakthrough viruses from parental virus 10 showed improved replication kinetics compared to the parental virus, suggesting that increased replication allows for enough virus production to overcome aCA-Fc restriction. These data suggest that the targeted region by aCA-Fc is constrained and not susceptible to escape mutations. aCA-Fc was also effective against LEN escape mutant variants and, importantly, against a wide variety of HIV-1 subtypes occurring around the world. Our results strongly suggest that capsid-targeting biologics are effective HIV-1 replication inhibitors with a very broad specificity and can be used in different antiretroviral strategies.

The biologic used in this study inhibits HIV-1 replication through degradation of Gag/CA. This biologic is composed of a single-domain Llama VHH (nanobody), which binds Gag/CA with high affinity, fused to human IgG1 Fc [40]. The latter will be recognized by the intracellular Fc-receptor TRIM21, as part of an intrinsic defence mechanism [36,37]. Upon binding, the complex will be sent to the proteasome for degradation [41]. We have previously shown that aCA-Fc is effective in inhibiting production of Gag at later stages of viral replication [35] and further demonstrate the robustness of the biologic here by expanding to primary isolates and different subtypes.

The epitope for the biologic is located in a conserved region in capsid (amino acid region 196–205 and 211–218 in CA region of reference HXB2D) [40]. Sequence analysis reveals some variance in group M in the first part of the epitope at position 200 and 203 [8]. These were also found in mutational analyses, with T200S but not K203A mutation yielding infectious virus [22,23]. Other mutations that were found here were D197A/N, A204G and T216A, with only the latter two yielding infectious virus but with low natural variation [23]. Therefore, these might be likely positions for potential capsid mutations required to adapt to the presence of aCA-Fc. Interestingly, in the original description of the anti-p24 nanobody 59H10, Gray et al. show lower levels of binding against one isolate from subtype D, which contains an I201L substitution in the region targeted by the nanobody that could explain this lower binding [40].

The amino acid changes we found in the breakthrough variants were not located in the region targeted by the biologic. Instead, one amino acid change was found in the CA, and one change was found in the nucleocapsid (NC) domain of Gag. For both breakthrough variant 4A and 4B there was a V83L amino acid change in the CA region, which is located at the end of helix 4 CA [19,42]. A leucine (L) at this position is a natural occurring variation (PMID: 19343217 [43]), so it is unlikely to be involved in adaption to aCA-Fc, in line with previous research demonstrating no effect on viral fitness from this substitution [44]. For variants 10A/B/C, there was a change at position 8 in the NC domain (p7) in a stretch of basic residues just upstream of the first zinc finger (ZF) motif. This region is involved in viral RNA packaging and gag–gag multimerization during viral assembly [45]. Changes to the basic residues near these ZF motifs could have an effect on the efficiency of viral assembly and thereby facilitate adaptation.

Changes in amino acids were mainly found in three regions of MA. For variants 4A and 4B, these changes were located in MA region 52–69. This region is essential for membrane anchoring and virus particle assembly, primarily through MA trimerization and interactions with lipids [46,47,48]. Deletion of this region decreases virus assembly, but specific mutations in this region, in combination with other MA mutations, have been shown to rescue Env incorporation and replication [49,50]. Furthermore, in breakthrough variants 10A, 10B and 10C, various amino acid changes were found in MA region 114–124. This region is located in helix 5 of MA, close to the CA N-terminal domain, and does not seem to be critical for Gag assembly and Env incorporation, although deletion of this region affects viral replication [42,48,51,52]. Lastly, variant 10D also showed changes in MA region 30–40, which is part of the highly basic region (HBR), a critical functional MA domain which is essential for binding cellular proteins and lipids [53,54]. Deletion of this whole region does not affect virion production, although mutant viruses were non-infectious [49]. Specifically, residue 30 has previously been noted as a crucial residue in MA and associated with changes in viral fitness [42,55,56]. Residue 35 was shown to be located in the trimer–trimer interface, likely stabilizing this interface and the capsid lattice, and specific mutations at this position caused a defect in Env binding [57,58]. Therefore, amino acid change Q30H in the HBR, and potentially E40K in breakthrough variant 10D, could increase binding of Gag to membrane by increasing the amount of basic residues in the HBR, a phenomenon reported before [59]. Taken together with mutation V35L, the amino acid changes in MA of breakthrough variants could result in less Env binding and increased and stronger Gag binding to the membrane, thereby avoiding aCA-Fc degradation and resulting in the faster replication kinetics we observed when compared to the parental virus. Even though we found various amino acid changes in the different breakthrough variants, none were universal among all variants, indicating that either different changes in Gag or amino acid changes outside of Gag could potentially lead to breakthrough. Moreover, no mutations were located in the region targeted by the biologic, suggesting a high barrier for mutations to arise that specifically hinder the binding of the biologic to Gag. True escape from the biologic is potentially hindered by the genetic constraints inherent to CA [60]. Rather, faster replication and continued sensitivity to aCA-Fc of breakthrough variants suggest that adaptation is likely due to a shift in replication kinetics which potentially allows for enough Gag in the presence of aCA-Fc to continue viral replication. This could also explain why virion-associated aCA-Fc was still detected in the supernatant of cells infected with breakthrough variants 10A–D, as it demonstrates that these viruses did not fully escape aCA-Fc binding.

HIV-1 exhibits high genetic diversity, classified into various subtypes. The heterogeneity between these subtypes complicates treatment due to differences in susceptibility to antiretroviral agents and emergence of resistance mutations. Even though there are distinct regional prevalence patterns between subtypes, subtype C is the most prevalent, followed by subtype A [3,4]. However, most HIV-1 pre-clinical studies have focused on subtype B characterization and treatment, which is dominant in Western and Central Europe, as well as Latin and North America [4]. Therefore, it is important to extend the characterization of HIV-1 and search in antiviral agents to the non-B subtypes [61]. Here we demonstrate that aCA-Fc is effective across HIV-1 subtypes from all around the world, despite natural sequence variation observed in the region of capsid targeted by aCA-Fc.

While these results provide important insights into the use of anti-capsid biologics, several limitations should be considered. Firstly, the anti-capsid biologic operates intracellularly, which means that delivery into the cell will be a challenge. Delivery could be achieved through different methods, like lipid nanoparticles [62]. Previously, purified aCA-Fc was successfully transferred into cells using a lipid-based formulation, resulting in capsid degradation in vitro [35]. Moreover, the ease of modification of these biologics allows for the addition of cell-targeting signals, which could help them reach specific cell populations [63,64]. However, these methods should be thoroughly tested before they can be employed in a clinical setting. One example of therapeutic use for this biologic could be to inhibit viral replication or transmission during activation of the HIV-1 reservoir.

Another limitation is that mutations of breakthrough variants were only identified in the *gag* gene. Amino acid changes in other regions may also increase replication kinetics and thus support breakthrough replication. This is supported by the observation that the mutations only occurred in the parallel cultures of two specific viruses, indicating that there might be something in the backbone of these viruses that makes them more susceptible to breakthrough under the selection pressure of aCA-Fc.

Here we have shown that the anti-capsid biologic aCA-Fc is able to recognize different HIV-1 subtypes occurring worldwide including dominant subtype C. The biologic has a high barrier to viral escape, and those viral variants that break through remain sensitive to aCA-Fc but are able to break through by enhancing replication kinetics. These data support an important role for anti-capsid inhibitors or biologics as potential drugs in antiretroviral therapies due to breadth and resistance to viral escape.

## 4. Materials and Methods

### 4.1. Cell Isolation and Culture

Human embryonic kidney cells containing the mutant version of the SV40 large T-cell antigen (HEK293T cells, ATCC CRL-3216) were cultured at 37 °C, 5% CO_2_ in Dulbecco’s Modified Eagle Medium (DMEM, Thermo Fisher Scientific, Waltman, MA, USA) containing 10% fetal calf serum (FCS, Sigma-Aldrich, Burlington, MA, USA), penicillin (100 U/mL) and streptomycin (100 µg/mL).

U87 cells expressing CD4 and CCR5 (U87.CD4.CCR5 [65]) were transduced with a HIV-1 based-lentiviral expression vector pLV-hEF1a-IRES-GFP containing the aCA-Fc sequence or an empty vector control, under the human Elongation Factor 1 alpha promotor, with an IRES and GFP sequence downstream as described before [35]. Cells were producing an average of 55.6 ± 18.6 pg of aCA-Fc as determined by binding ELISA. U87.CD4.CCR5 cells were cultured in Iscove’s Modified Dulbecco Medium (IMDM, Thermo Fisher Scientific, Waltman, MA, USA) supplemented with 10% FCS, penicillin (100 U/mL) and streptomycin (100 µg/mL) and treated once a month with G418 (Invitrogen, Carlsbad, CA, USA) and puromycin (Sigma-Aldrich, Burlington, MA, USA). Cells were maintained at 37 °C, 10% CO_2_.

Peripheral blood mononuclear cells (PBMCs) were isolated from the buffy coats of healthy blood donors, pooled and cryopreserved. After thawing, the PBMCs were cultured in IMDM (Thermo Fisher Scientific, Waltman, MA, USA) supplemented with 10% FCS, penicillin (100 U/mL), streptomycin (100 µg/mL), ciproxin (5 µg/mL), recombinant interleukin 2 (rIL2, 20 U/mL) and polybrene (5 µg/mL). Cultures were maintained at a cell density of 5 × 10^6^ cells/mL in a humidified 10% CO_2_ incubator at 37 °C.

### 4.2. Production of HIV-1 in Peripheral Blood Mononuclear Cells (PBMCs)

HIV-1 isolates 99KE_KNH1088 (subtype A), 00KE_KSM4030 (subtype A), 91US_4 (subtype B), 92FR_BX08 (subtype B), 94IN_20635-4 (subtype C), 00TZ_A125 (subtype C), 98UG_57128 (subtype D), 00KE_NKU3006 (subtype D), 90TH_CM235 (subtype CRF01_AE), 96TH_NI1046 (subtype CRF01_AE), 91DJ_263 (subtype CRF02_AG) and 01CM_0008BBY (subtype CRF02_AG) were obtained through the NIH HIV reagent programme. CCR5-using primary HIV-1 variants (subtype B) were previously described [38,39]. HIV-1 variants were grown according to previously described protocol [66]. In short, donor peripheral blood mononuclear cells (PBMCs) were stimulated for 2 days with phytohemagglutinin (PHA; 1 μg/mL) in IMDM supplemented with 10% FCS, penicillin (100 U/mL), streptomycin (100 U/mL) and ciproxin (5 µg/mL) at 5 × 10^6^/mL cells. Next, 5 × 10^6^ stimulated PBMCs were combined with the different viruses, shaken for 2 h at 37 °C, and then transferred to a T25 culture flask in 5 mL of IMDM supplemented with IL-2, 10% FCS, penicillin, streptomycin and ciproxin. After 7 days, a sample of the culture was tested for p24 levels, 1/3 of the culture was discarded, and 5 × 10^6^ more stimulated PBMCs were then added and cultured for another 7 days. The supernatant of positive cultures was harvested and stored at −80 °C.

Virus titers were determined by infecting TZM-bL cells and calculating the TCID_50_. Luminescence was determined after 3 days using an in-house luciferase assay: 25 μL of luciferase substrate (0.83 mM ATP, 0.83 mM d-luciferin (Duchefa Biochemie B.V., Haarlem, The Netherlands), 18.7 mM MgCl_2_, 0.78 μM Na_2_H_2_P_2_O_7_, 38.9 mM Tris (pH 7.8), 0.39% (*v*/*v*) glycerol, 0.03% (*v*/*v*) Triton X-100, and 2.6 μM dithiothreitol) was added directly to the culture medium in each well. Luminescence was measured using a luminometer (Berthold Technologies, Bad Wildbad, Germany).

### 4.3. Viral Infection Assay

U87.CD4.CCR5 cells stably expressing the aCA-Fc or an empty vector control were inoculated with different HIV-1 variants at varying multiplicity of infection (MOI). Samples were taken after seven days of infection by inactivating supernatant or cell lysate in 0.1% Triton X-100 (final concentration). Replication was analyzed using an in-house p24 ELISA [67].

Replication kinetics of HIV-1 was determined on U87.CD4.CCR5 cells stably expressing the aCA-Fc or empty vector. To this extent, cells were cultured in a 24-well plate at a density of 8 × 10^3^ cells per well. The next day the cells were inoculated with 100 TCID_50_ of HIV-1, and the medium was refreshed 24 h later. On each following day viral replication in the supernatant was determined using an in-house p24 ELISA [67].

### 4.4. Production and Transfection of LEN-Resistant Mutants

N74D and Q67H mutations were generated by site-directed mutagenesis of Tat-independent Gag–Pol expression vector pMDL/pRRE [68] using the CloneAmp HiFi PCR Premix (TakaraBio, San Jose, CA, USA) and confirmed by sequencing. Mutant constructs were co-transfected with a Rev-expressing construct (pRSV-Rev [68]), as well as an aCA-Fc-expressing construct (pLV-hEF1a-aCA-Fc-IRES-GFP [35]) or an empty vector control (pLV-hEF1a-IRES-GFP), in HEK293T cells using the calcium phosphate method. Briefly, 1.87 µg of pRSV-Rev, 2.43 µg of pMDL plasmid, and 3.77 µg of aCA-Fc or control DNA was diluted in 0.042 M of HEPES containing 0.2 M of CaCl_2_ and mixed with an equal volume of 2× HEPES-buffered saline (HBS). After incubating for 15 min, the mixture was gently added to HEK293T cells in a 6-well plate, and the cells were incubated overnight at 37 °C in a humidified 3% CO_2_ incubator. The following day, the culture medium was replaced, LEN (1 nM or 100 nM) was added, and then cell cultures were maintained at 10% CO_2_ at 37 °C. Three days after transfection, the amount of produced p24 was determined by in-house p24 ELISA [67].

### 4.5. In Vitro Evolution of HIV-1 Against aCA-Fc

In vitro evolution experiments were performed, adapted from a previously described protocol [69]. In brief, U87.CD4.CCR5 cells were plated in a 24-well tissue culture plate at 40 × 10^3^ cells/well and inoculated with 1 mL of HIV-1 (>500 ng p24/mL). For each viral variant, 4 replicates were cultured in parallel. Each following week, viral replication in the supernatant is determined by p24 ELISA [67], and the culture supernatant is transferred to a fresh U87.CD4.CCR5 coculture containing an increasing proportion of U87.CD4.CCR5 transduced to continuously express aCA-Fc. (90%, 95%, 99% for weeks 2, 3 and 4 respectively) and is maintained at 99% for the remaining weeks (up to 15 weeks) until viral breakthrough is detected in the culture supernatant. Cultures that tested positive for p24 were transferred to fresh cultures containing 100% U87.CD4.CCR5 cells expressing aCA-Fc and then cultured for 7 more days, after which culture supernatant was collected for DNA isolation and sequencing.

### 4.6. RNA Isolation, PCR and Sequencing

Total RNA was isolated using a QIAamp Viral RNA Mini Kit (QIAGEN, Venlo, The Netherlands). cDNA was produced by incubating the RNA with a random primer mix (Promega, Madison, WI, USA) at 70 °C for 5 min, followed by incubation on ice for 5 min and then reverse transcription using M-MLV Reverse Transcriptase (Promega, Madison, WI, USA), with incubation of 1 h at 42 °C. Gag PCR amplification of the produced cDNA was performed using primers Gag-BstHII (fw) (5′-TGCTGAAGCGCGCACGGC-3′) and GAG-ApaI (rev) (5′-AACGTCCCGGGGATCCTT-3′), as well as a nested PCR with p24-5E (fw) (5′-AGCAATCAGGTCAGCCAAAATTAC-3′) and C2 (rev) (5′-GGGTACAAAAGTCGTAATAGTCT-3′) using GoTaq^®^ DNA Polymerase (Promega, Madison, WI, USA). PCR products were purified using ExoSAP-IT (Thermo Fisher Scientific, Waltman, MA, USA) according to the manufacturer’s protocol. Sequencing of Gag was performed using a BigDye Terminator v1.1 Cycle Sequencing kit (ABI Prism, Applied Biosystems, Foster City, CA, USA) according to the manufacturer’s protocol, using the following amplification cycles: 5 min at 95 °C, 30 cycles of 30 s at 95 °C, 30 s at 50 °C, 2 min at 60 °C, followed by a 5 min extension at 60 °C and subsequent cooling to 4 °C. Next, plates were washed and then a mix of intact and monomeric HIV-1 Gag/CA from the detergent-treated culture supernatant from HIV-1 infected cells were added, followed by 2 h of incubation at 37 °C.

### 4.7. Fc-Specific Binding ELISA

To determine the aCA-Fc concentration of U87.CD4.CCR5 cells, an Fc-specific ELISA was performed, adapted from an in-house p24 ELISA [67]. In short, high-binding plates (Nunc MaxiSorp™ flat-bottom, Thermo Fisher Scientific, Waltman, MA, USA) were coated overnight at 4 °C with a mouse anti-p24 antibody in PBS. Next, a mix of intact and monomeric HIV-1 Gag/CA from detergent-treated culture supernatant from HIV-1 infected cells were added and incubated at 37 °C for 2 h. Then, cell lysate from cells expressing aCA-Fc was added. After a 2 h incubation at 37 °C, the plates were incubated for 1.5 h at 37 °C with a secondary antibody conjugated to horseradish peroxidase (goat anti-human AffiniPure F(ab’)2 Fragment (Jackson ImmunoResearch, West Grove, PA, USA)) diluted 1:10,000 in PBS with 5% NGS and 0.01% Tween. Lastly, binding was visualized using the substrate solution (0.1 M NaAc + 0.1 M citric acid + 0.84 mg/mL TMB + 0.01% H_2_O_2_). A total of 2 M of H_2_SO_4_ was used to stop the reaction, and optical density was measured using a 450 nm filter. The plates were washed between all steps. A serial dilution of purified aCA-Fc was used as a standard curve.

Similarly, to determine the presence of Gag/CA-bound aCA-Fc in the supernatant of infected cells, the supernatant of U87.CD4.CCR5 cells expressing aCA-Fc infected with parental or breakthrough variants and inactivated in 0.1% Triton X-100 (final concentration) was incubated on coated plates, followed by incubation with the secondary antibody and subsequent addition of substrate.

### 4.8. Data Analysis

Nucleotide sequences of primary isolates were combined and aligned using ClustalW in the software package BioEdit v.7.0.9.0 (Hall [70]) and then edited manually.

Statistical analyses were performed using GraphPad Prism v10.2 (GraphPad software Inc., San Diego, CA, USA). Multiple comparisons of unpaired grouped data were determined using a two-way ANOVA test with the Šidák multiple comparisons test. Statistical significance was set at *p* < 0.05 (ns = not significant, * *p* < 0.05; ** *p* < 0.01, *** *p* < 0.001).

## Data Availability

The original contributions presented in this study are included in the article/Supplementary Material. Further inquiries can be directed to the corresponding authors.

## Supplementary Materials

The following supporting information can be downloaded at: https://www.mdpi.com/article/10.3390/ijms27156883/s1.

## Abbreviations

The following abbreviations are used in this manuscript:

HIV-1	Human immunodeficient virus 1
ART	Antiretroviral therapy
PWH	People with HIV
CRF	Circulating recombinant form
CA	Capsid
MA	Matrix
NC	Nucleocapsid
AA	Amino acid
LEN	Lenacapavir
TRIM21	Tripartite motif 21
ZF	Zinc finger
VHH	Variable heavy-chain domain of heavy-chain only antibody
HBR	Highly basic region
PBMC	Peripheral blood mononuclear cells
MOI	Multiplicity of infection
