# Supplementary material for: Capsid-Targeting Biologic Achieves Broad HIV-1 Neutralization with a High Barrier to Resistance
Source: Int J Mol Sci. 2026 Aug 1;27(15):6883. doi: 10.3390/ijms27156883 (PMC13467310; doi:10.3390/ijms27156883)
Supplement: Supplementary file 1 [file ijms-27-06883-s001.zip › ijms-4370622-supplementary.pdf]

Supplementary Figure S1: *In vitro* evolution of primary HIV-1 isolates without breakthrough

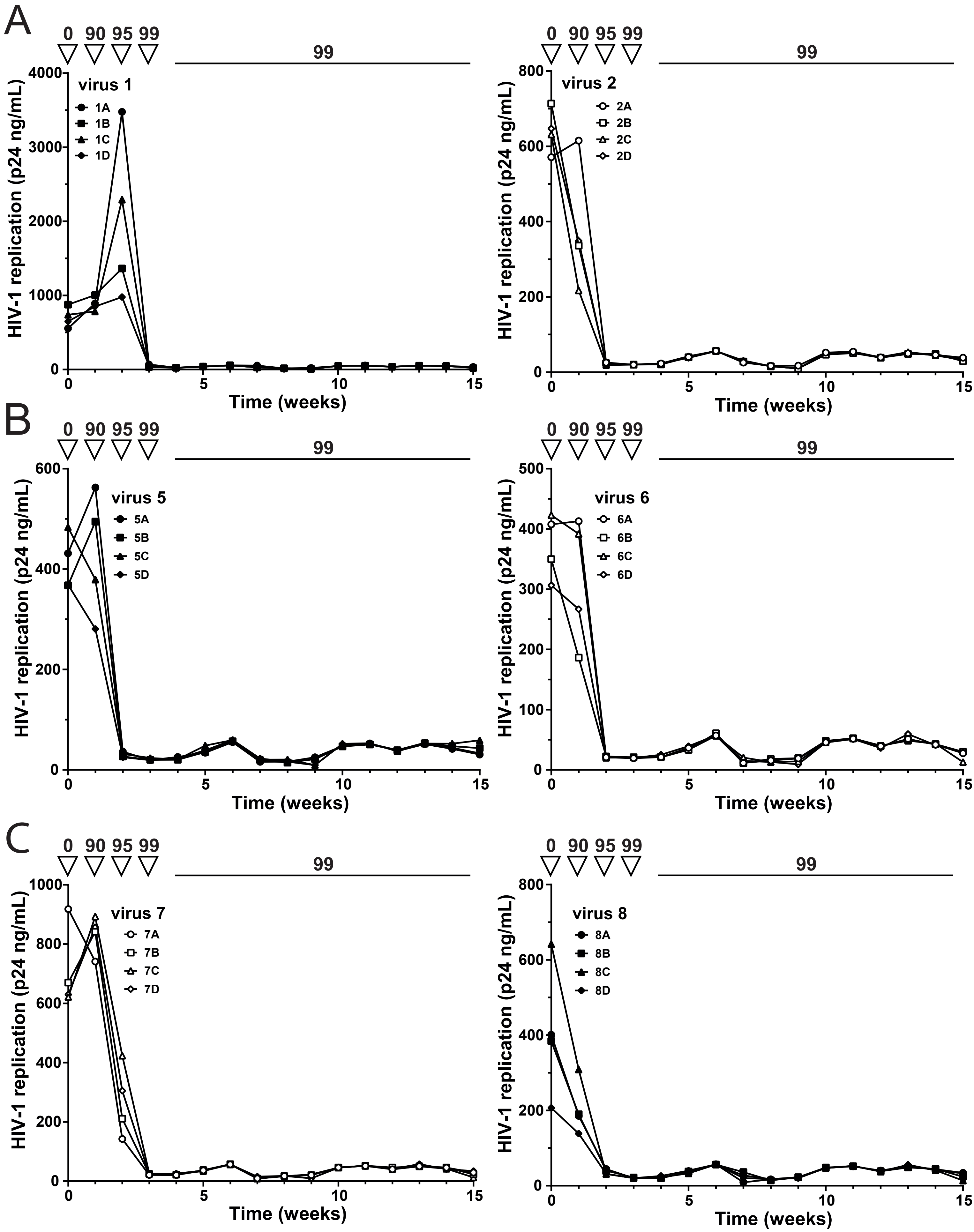

Figure S1: Replication kinetics of primary CCR5-variants from PLWH-1 **(A)**, PLWH-3 **(B)** and PLWH-4 **(C)** during *in vitro* culturing in U87.CD4.CCR5 cells with increasing percentages of aCA-Fc-producing cells. Arrows on top indicate the percentage of U87.CD4.CCR5 cells with aCA-Fc.

Supplementary Figure S2: Gag sequences of primary isolates without breakthrough

```
HXB2D              MGARASVLSGGELDRWEKIRLRPGGKKKYKLKHIVWASRELERFAVNPGLLETSEGCRQILGQLQPSLQTGSEELRSLYN
parental virus 1   .............................QF..VI..................A.......E....A.K......K....
parental virus 2   .............................QF..VI..................A.......E....A.K......K....
parental virus 3   ...........Q..K..R...........R........................D......E....A.K...........
parental virus 5   ..............K.......................................D.........................
parental virus 6   ..............K..............Q.......................A..........................
parental virus 7   ..............K.......................................D.........................
parental virus 8   ..............K............R..........................Q......E..................
parental virus 9   ..............K..............Q.......................A..........................

HXB2D              TVATLYCVHQRIEIKDTKEALDKIEEEQNKSKKKAQQAA~~~~ADTGHSNQVSQNYPIVQNIQGQMVHQAISPRTLNAWV
parental virus 1   ..........K.DV......................QAA~~.A....N.S...........L...A....L.........
parental virus 2   ..........K.DV......................QAA~~.A....N.S...........L...A....L.........
parental virus 3   ..........K..V........E.............QAAASTA.G..N.S...........M..................
parental virus 5   .............V......V...............QAA~~~~....C.S...........L..................
parental virus 6   ..........K..V........E.V........R..QAQ...~.G..N.S...........M..................
parental virus 7   .............V......V...............QAA~~~~....C.S...........L..................
parental virus 8   .............V..........V...........QAA~~~~....NNS...........L..................
parental virus 9   ..........K..V........E.V........R..QAQ...~.G..N.S...........M..................

HXB2D              KVVEEKAFSPEVIPMFSALSEGATPQDLNTMLNTVGGHQAAMQMLKETINEEAAEWDRVHPVHAGPIAPGQMREPRGSDI
parental virus 1   ..........................................................L.....................
parental virus 2   ..........................................................L.....................
parental virus 3   ..I.......................................................L.......V.............
parental virus 5   ...........I..............................................L...Q...V.............
parental virus 6   ..I.......................................................L...Q...V......D......
parental virus 7   ...........I..............................................L...Q...V.............
parental virus 8   ..........................................................L.....................
parental virus 9   ..I.......................................................L...Q...V......D......

HXB2D              AGTTSTLQEQIGWMTNNPPIPVGEIYKRWIILGLNKIVRMYSPTSILDIRQGPKEPFRDYVDRFYKTLRAEQASQEVKNW
parental virus 1   ...............H...........................A....................................
parental virus 2   ...............H...........................A....................................
parental virus 3   ...........................................V...............................D....
parental virus 5   ...........................................V...............................D....
parental virus 6   ...........................................V....................................
parental virus 7   ...........................................V...............................D....
parental virus 8   .................................................K..............................
parental virus 9   ...........................................V....................................

HXB2D              MTETLLVQNANPDCKTILKALGPAATLEEMMTACQGVGGPGHKARVLAEAMSQVTNSA~~TIMMQRGNFRNQRKIVKC
parental virus 1   .......................G................................ANANA.............A...
parental virus 2   .......................G............................L...ANANA......D......A...
parental virus 3   ........................................S....I.......A....~~.M.......K..KRTI..
parental virus 5   ..........................................................~~.....K............
parental virus 6   ..........................................................~~.....K....S.......
parental virus 7   ..........................................................~~.....K............
parental virus 8   ........................................................TT~~S.............T...
parental virus 9   ..........................................................~~.....K....S.......
```

Capsid

aCA-Fc epitope

Figure S2: Gag sequences of primary isolates without breakthrough. Amino acids positions of capsid are marked (yellow), and the aCA-Fc epitope in HXB2D is indicated by underlining

Supplementary Figure S3: Gag sequences of parental and aCA-Fc breakthrough variants

```
HXB2D               MGARASVLSGGELDRWEKIRLRPGGKKKYKLKHIVWASRELERFAVNPGLLETSEGCRQILGQLQPSLQTGSEELRSLYN
parental virus 4    ...........Q..K..R...........R.....................K.A.......E..P.A.R...........
4A                  ...........Q..K..R...........R.......................T.......E....A.K...........
4B                  ...........Q..K..R...........R.......................T.......E....A.K...........
parental virus 10   ..............K..............Q.......................A........................F.
10A                 ..............K..............Q.......................A........................F.
10B                 ..............K..............Q.......................A........................F.
10C                 ..............K..............Q.......................A........................F.
10D                 ..............K..............H....LG.T.K.............A........................F.

HXB2D               TVATLYCVHQRIEIKDTKEALDKIEEEQNKSKKKAQ~~~QAAADTGHS~~NQVSQNYPIVQNIQGQMVHQAISPRTLNAW
parental virus 4    ..........K..V.......E..............~~~....G..N.SSS...........L.................
4A                  ..........K..V.......E..............~~~....G..N.SSS...........L.................
4B                  ..........K..V.......E..............~~~....G..N.SSS...........L.................
parental virus 10   ..........K..V.......E.V............QAQ...TG..S.~~S...........M.................
10A                 ..........K..V.......E.V.........R..QAQ....G..N.~~S...........M.................
10B                 ..........K..V.......E.V............QAQ...TG..S.~~S...........M.................
10C                 ..........K..V.......E.V............QAQ...TG..N.~~S...........M.................
10D                 ..........K..V.......E.V......I.....QAQ...TG..S.~~S...........M.................

HXB2D               VKVVEEKAFSPEVIPMFSALSEGATPQDLNTMLNTVGGHQAAMQMLKETINEEAAEWDRVHPVHAGPIAPGQMREPRGSD
parental virus 4    ....................A..............................................V............
4A                  ....................A......................................L.......V............
4B                  ....................A......................................L.......V............
parental virus 10   ...I.......................................................L..AQ...V......D.....
10A                 ...I.......................................................L..AQ...V......D.....
10B                 ...I.......................................................L..AQ...V......D.....
10C                 ...I.......................................................L..AQ...V......D.....
10D                 ...I.......................................................L..AQ...V......D.....

HXB2D               IAGTTSTLQEQIGWMTNNPPIPVGEIYKRWIILGLNKIVRMYSPTSILDIRQGPKEPFRDYVDRFYKTLRAEQASQEVKN
parental virus 4    ............................................V...............................D...
4A                  ............................................V...............................D...
4B                  ............................................V...............................D...
parental virus 10   ................S...........................V...................................
10A                 ................S...........................V...................................
10B                 ................S...........................V...................................
10C                 ................S...........................V...................................
10D                 ................S...........................V...................................

HXB2D               WMTETLLVQNANPDCKTILKALGPAATLEEMMTACQGVGGPGHKARVLAEAMSQVTNSATIMMQRGNFRNQRKIVKC
parental virus 4    .........................................S....I.....................K..KRTI..
4A                  .........................................S....I.....................K..KRTI..
4B                  .........................................S....I.....................K..KRTI..
parental virus 10   ................................................................K....D.......
10A                 ................................................................K............
10B                 ................................................................K....G.......
10C                 ................................................................K....G.......
10D                 ................................................................K............
```

Capsid

aCA-Fc epitope

Figure S3: Gag sequences of parental and aCA-Fc breakthrough variants. Amino acids positions of capsid are marked (yellow), and the aCA-Fc epitope in HXB2D is indicated by underlining

Supplementary Figure S4: Gag sequences for HIV-1 subtypes

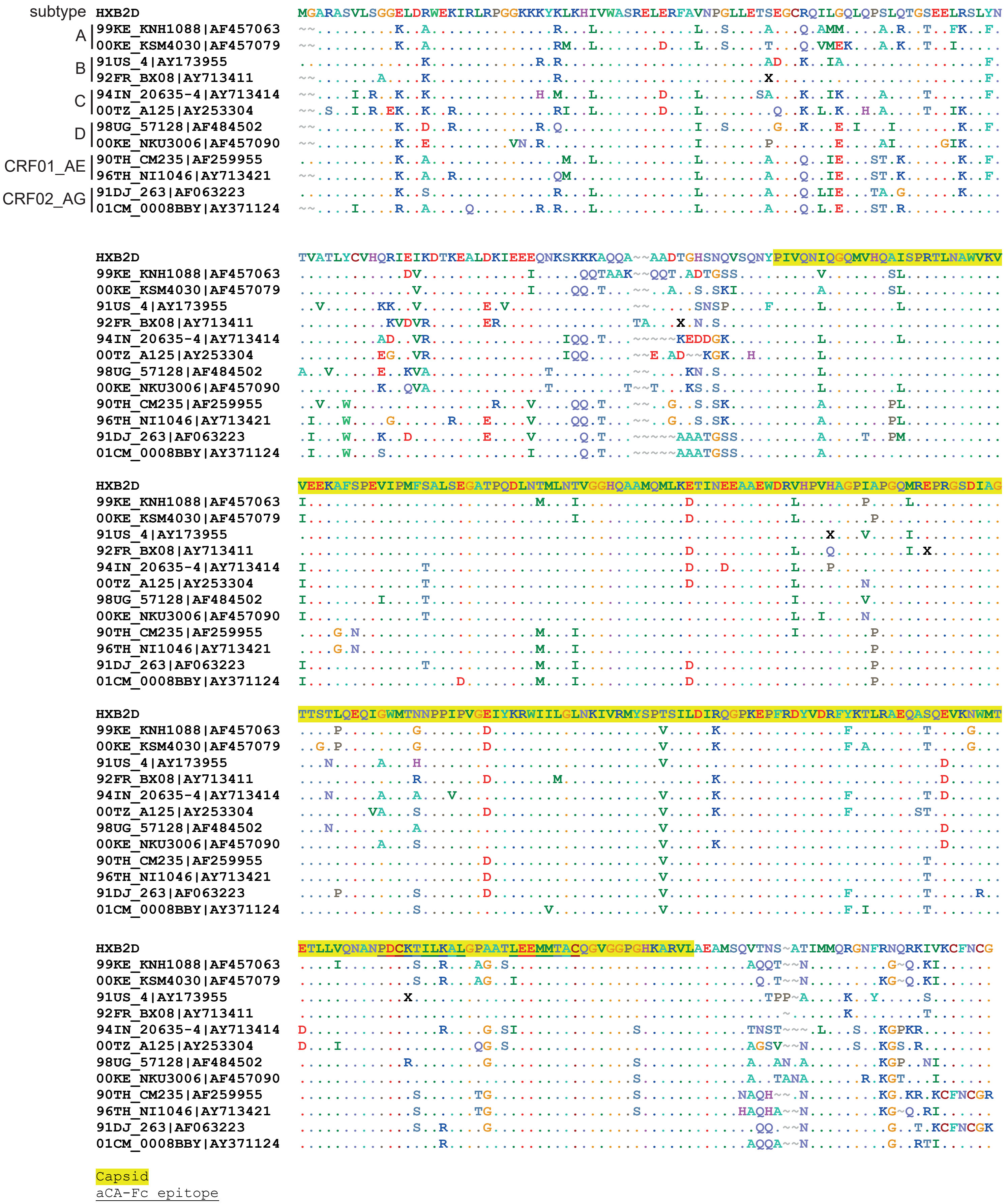

Figure S4: Gag sequences for subtype variants. Amino acids positions of capsid are marked (yellow), and the aCA-Fc epitope in HXB2D is indicated by underlining. For each subtype variant, isolate name and NCBI database entry number are indicated.
